# Data on the soilscape and vegetation properties at the key site in the NW Caspian sea coast, Russia

**DOI:** 10.1016/j.dib.2020.105972

**Published:** 2020-07-02

**Authors:** Ivan Semenkov, Maria Konyushkova, Ahmad Heidari, Yulia Nukhimovskaya, Galya Klink

**Affiliations:** a Lomonosov Moscow State University, Russia; b University of Tehran, Iran; c Severtsov Institute of Ecology and Evolution of the Russian Academy of Sciences, Russia; d Institute for Information Transmission Problems (Kharkevich Institute) of the Russian Academy of Sciences, Russia

**Keywords:** Potentially toxic elements, Entisols, Vertical distribution, Low energy environments, Recent fine-textured soils, AMS-dating, Partition, Heavy metals

## Abstract

Research on the environment in recent soils is important to understand geochemical processes in coastal landscapes and the rate of pedogenesis. In this article, we present original data on Gleyic Solonchaks (Loamic) and vegetation described at the eastern part of the Terek–Kuma lowland (Northern Dagestan, Russia). At the key site of 45 × 30 m released from water 293±13 years calBP, we described vegetation at 345 plots of 2 × 2 m (4 m^2^) and soil properties in 58 auger holes and 2 pedons, the latter characterizing a typical microhigh with Tamarix and a microlow with saltworts. The flora of the sites amounts to 32 species (predominantly, halophytes) belonging to 11 families. Shrubs represented by tamarixes are the dominant. Under their crowns, dense herb and grass microcommunities with a predominance of tall *Puccinellia gigantea* occur. Sparse stunted halophytic plants (*Petrosimonia, Frankenia, Puccinellia*) occupy open habitats between shrubs. In soil water extracts from auger holes (696 samples in total), we measured electrical conductivity (EC) and pH. In 49 soil samples from pedons, we described particle size distribution, total concentration of macro elements (Al, Ca, Fe, K, as well as Mg, Mn, P, Ti, and Si) and trace elements (As, Co, Cr, Cu, Ni, Pb, Sr, and Zn), EC, pH, basicity (HCO_3_^–^ and CO_3_^2–^) as well as the content of cations (Ca^2+^, Mg^2+^, Na^+^, and *K*^+^) and anions (SO_4_^2–^ and Cl^–^) in soil water extracts. Gleyic Solonchaks (Loamic) with bulk density of 1.35±0.12 g/cm^3^ (mean and standard deviation) contain SiO_2_ 69±8%> Al_2_O_3_ 11.8 ± 3.5 and CaO 7.5 ± 2.5%, Fe_2_O_3_ 3.6 ± 1.4%, K_2_O 2.0 ± 0.3 and MgO 1.9 ± 0.4%> TiO_2_ 0.62±0.25%> P_2_O_5_ 0.14±0.06% and MnO 713±268 mg/kg> Sr 481±262 mg/kg > Cr 79±9 mg/kg > *V* 76±36, Zn 68±31, Cu 62±10, and Ni 50±17 mg/kg, Co 32±6 mg/kg> Pb 11±6 mg/kg> As 5.6 ± 1.4 mg/kg. The particle-size distribution is (WRB system,%): clay 13±5, fine silt 34±12, coarse silt 30±18, as well as very fine sand 11±10, fine sand 7.3 ± 10.5, medium sand 3.5 ± 5.8, coarse sand 0.9 ± 3.2, and very coarse sand 0.08±0.31 (*n* = 38). Soil water extract has EC 9.4 ± 4.1 dSm/m (soils: water ratio of 1:2.5, *n* = 713), contains Na^+^ 15.9 ± 7.0 > Ca^2+^7.3 ± 5.0 and Mg^2+^ 7.3 ± 3.1 > *K* ^+^ 0.30±0.20 cmol(eq)/kg, as well as Cl^–^ 15.7 ± 7.3 and SO_4_^2–^ 14.6 ± 7.9 > HCO_3_^–^ 0.55±0.15 > CO_3_^2^^–^< 0.01 cmol(eq)/kg, and has pH 7.9 ± 0.3 (soils: water ratio of 1:5, *n* = 21). In soil paste, pH is 8.3 ± 0.2 (*n* = 461). Data obtained can be used for more confident identification of pollution sources and pollutants’ migration routes and more effective conservation and remediation of human-affected soils at the Caspian Sea coast.

**Specifications table**

**Table:** 

Subject	Environmental science (General)
Specific subject area	Environmental Chemistry, Earth Sciences, Biology, Soil Science, Botany
Type of data	Table Chart Graph Figure
How data were acquired	Radiocarbon dating of total organic carbon from soil samples was performed in the Center for Collective Use “Laboratory of Radiocarbon Dating and Electron Microscopy” (Institute of Geography RAS, Russia) and the Center for Applied Isotope Studies of the University of Georgia (USA) using radiocarbon calibration program ‘Calib Rev 7.1.0′ [1]. Elemental composition of soil samples was obtained using a Spectroscan Max-GV X-Ray fluorescence spectrometer (Russia). Particle-size distribution was measured using an ‘Analysette 22 Nano Tech’ equipment (Germany). In the soil water extracts, electrical conductivity and pH (as well as in paste) were measured at Hanna Combo 98,130 (Germany), content of Cl–was determined by means of titration with AgNO_3_, content of Ca^2+^ and Mg^2+^by means of complexometric titration with EDTA, content of Na^+^ and *K*^+^ via flame photometry. Phylogenetic tree of species found at the key site was built on a basis of a phylogenetic tree using scripts and instructions from [2].
Data format	Raw Analyzed
Parameters for data collection	Data were collected during field and laboratory works. A total of 745 soil samples were collected from the 58 auger holes and 2 pedons (Fig. 1) at the eastern part of the Terek–Kuma lowland (NE Dagestan, Russia).
Description of data collection	Before auger holes drilling, we described vegetation throughout the whole key sites along 30-m long transects located at a distance of 2 m from each other. The interval of geobotanical descriptions along transects was 2 m. In total, 345 descriptions were made (Table S1). Auger holes are drilled according to semi-regular grid with an interval of 1 to 5 m from each other. From auger holes, soil samples (200 – 300 g) were taken from a depth 0–5, 5–10, 10–20, 20–30, 30–50, 50–70, 70–100 cm. At the typical microhigh and microlow, we dug two soil pits. From pits, soil samples (300 – 500 g) were collected from Ayz, Bgz and Cgz horizons from a depth 0 – 3 m.
Data source location	Institution: Lomonosov Moscow State University Region: Dagestan republic Country: Russia The sampling area is located on the eastern part of the Terek–Kuma lowland (NW Caspian Sea coast). GPS coordinates of the corners of the sampling area (fig. 1): A. N 44.55287, E 46.67680 B. N 44.55310, E 46.67696 C. N 44.55295, E 46.67747 D. N 44.55270, E 46.67733 Coordinates of the pits: 05–17. N 44.5529, E 46.67688 (microhigh), 06–17. N 44.55281, E46.67717 (microlow) Primary data sources for the construction of the phylogenetic tree of species found at the key site were taken from [2].
Data accessibility	With the article
Related research article	I. Semenkov, M. Konyushkova, A. Heidari, E. Nikolaev, Chemical differentiation of recent fine-textured soils on the Caspian Sea coast: a case study in Golestan (Iran) and Dagestan (Russia), Quaternary International. In Press. https://doi.org/10.1016/j.quaint.2020.05.053

## 1 Value of the data

Data could be used for the assessment of floristic richness, soil salinity variability and the content of different chemical elements at the recent environments, as well as for adaptation of land use to the conditions of the Caspian Sea coast with a constant change in sea level.

Data may be useful for farmers, researchers and practitioners to adapt and mitigate soil salinity effects, for geochemists to evaluate migration of salts and potentially toxic elements in soilscapes and for botanist to characterize flora of the recent sea coast.

Data will be important for further estimation of soil and plant cover evolution in the recent Caspian Sea coast.

### 1.1 Data description

The Caspian Sea coast is the rapidly changing ecosystem due to a sea level fluctuation [3]. The present-day Caspian Sea sea level is –28.0 m a.s.l. At its recent coasts, the redistribution of salts and other chemical compounds is occurring. In order to track the processes of the formation of soil and vegetation patterns at the yearly stages of ecosystem formation, we performed a comprehensive and detailed soil, topography, and vegetation survey [5].

**Fig. 1:**
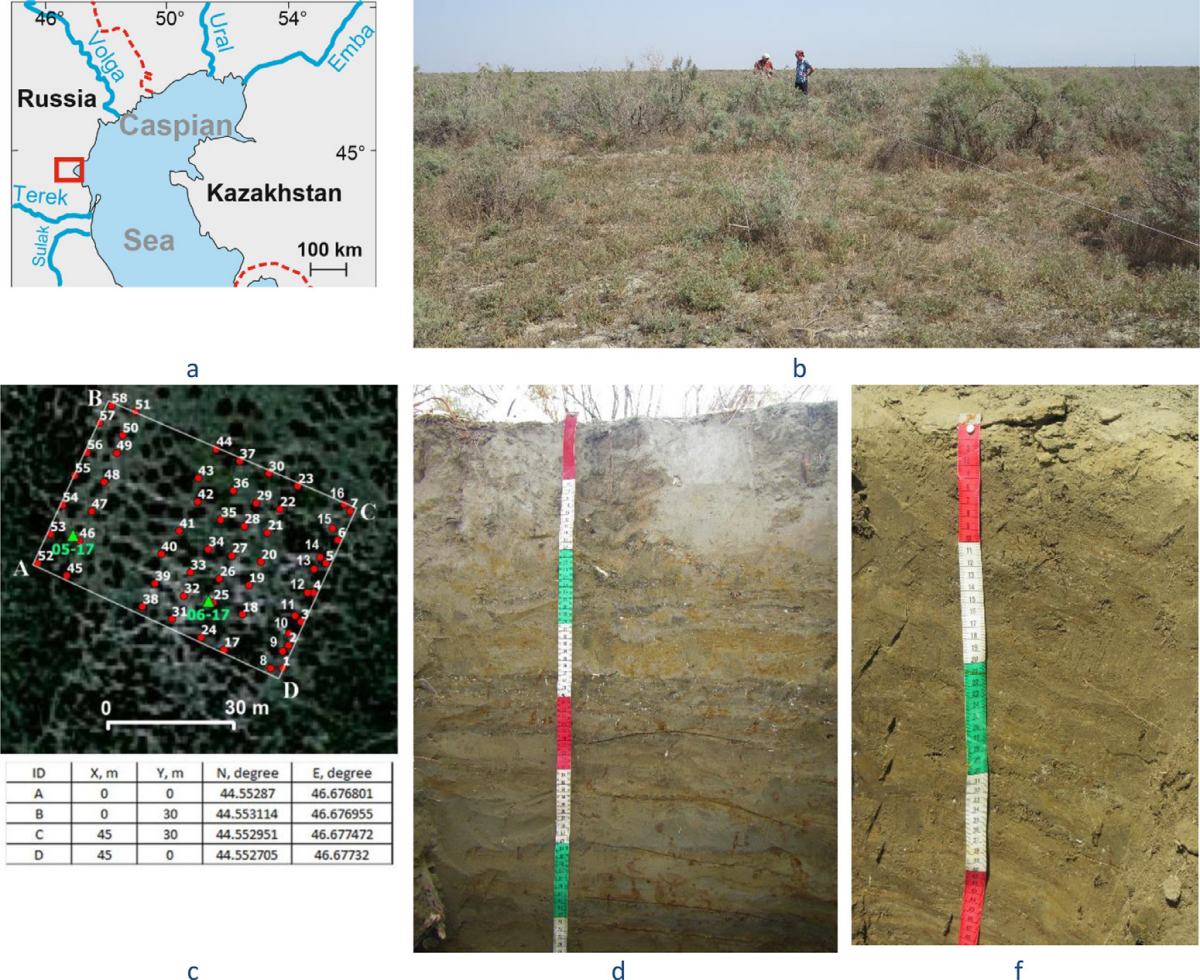
Location of key site (a). Vegetation (b). Location of auger holes and soil pits (c): 05–17– at microhigh (d), 06–17 – at microlow (e).

Data were collected at the flat key site of 45 × 30 m (–25.8 m a.s.l. according to Kronstadt tide gage) located to the NW part of the Caspian Sea and having flat topography and fine-textured deposits (Fig. 1). Groundwater level is 2.8 m (in September 2017 and 2018) with the solute concentration (predominantly, sodium chloride) 44–48 g/l [4].

In this paper, we presented data on 745 soil samples collected from 58 auger holes and 2 pedons sampled twice (during spring and autumn) and data on the geobotanical descriptions at 345 plots of 2 × 2 m (4 m^2^). According to GNSS-data [6], the amplitude of microtopography is 15 cm.

**Fig. 2:**
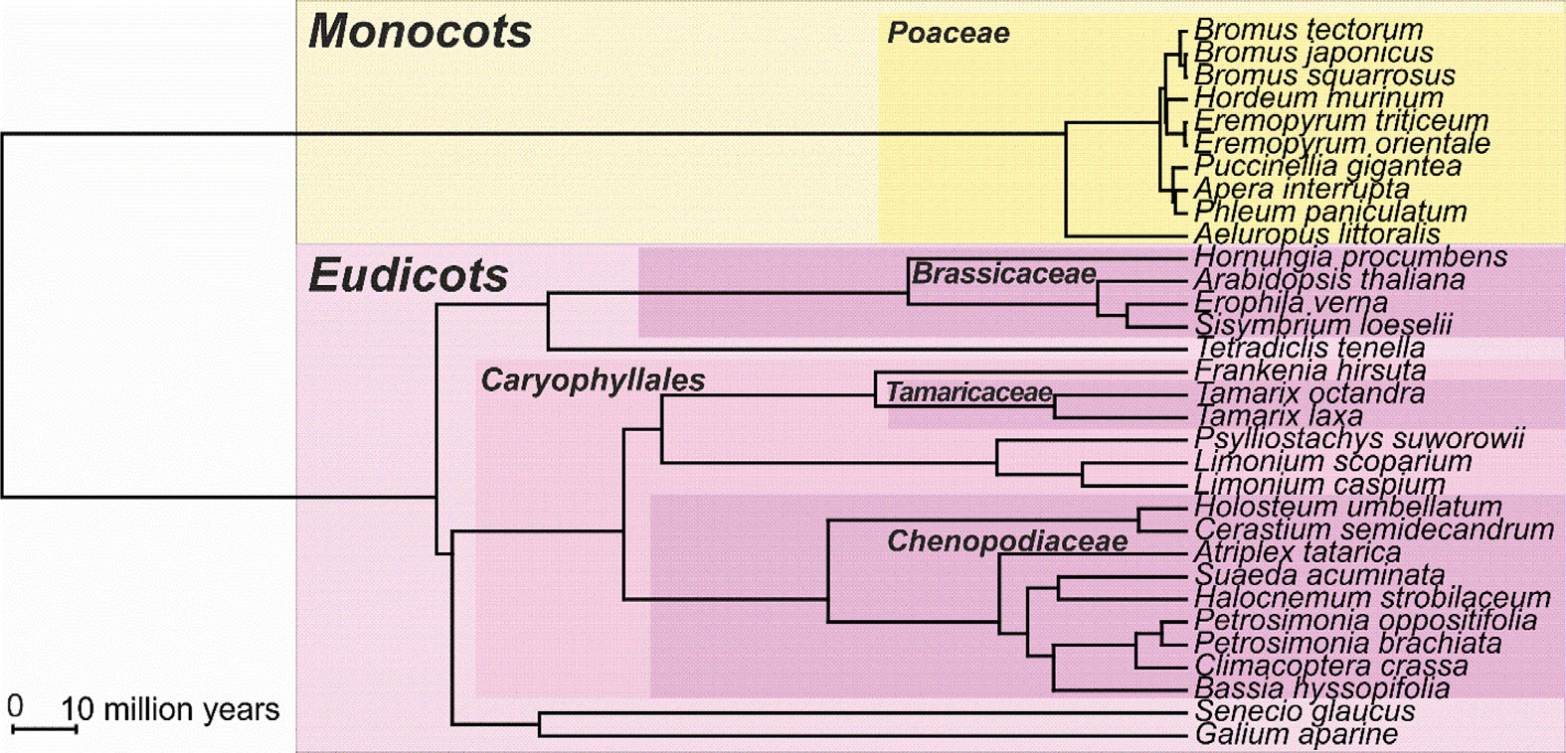
Evolutionary tree of species from the key site.

At the sampled Gleyic Solonchaks (Loamic) [4], [5], [6] formed from the saline sediments, the vegetation is a sparse degrading (deteriorating) community of 1–1.5 m high tamarix (*Tamarix octandra* mixed with some *T. laxa*) with annual saltworts (*Petrosimonia brachiata, P. oppositifolia, Suaeda acuminata*), *Frankenia hirsuta* and *Puccinellia gigantea* in the lower tier with some spring and early summer ephemeral plants. In the lower tier, two microgroups are clearly distinguished. Open habitats are occupied by sparse stunted halophytic plants (*Petrosimonia, Frankenia, Puccinellia*). Under the crowns of the bushes there are dense herb and grass microcommunities with a predominance of tall *Puccinellia gigantea* and a few *Limonium caspium* and L. *scoparium, Psylliostachys spicata* whereas saltworts and *Frankenia* are absent here. The flora of the site amounts to 32 species belonging to 11 families (Fig. 2).

Most species are halophytes. Shrubs are the dominant species and are represented by tamarixes. Semishrubs are represented by *Frankenia hirsuta*. There are 5 herbaceous perennials and 7 species of annual herbs belong to the *Chenopodiaceae* family. The most numerous biomorphotype is ephemeral plants (Table 1).

**Table 1:** Angiosperms flora of the site under consideration.

Clade [7]	Order	Family	Species	Type of vegetation
*Eudicots*	*Asterales*	*Asteraceae*	*Senecio noeanus*	Ephemeral plants
*Eudicots*	*Brassicales*	*Brassicaceae*	*Arabidopsis thaliana*	Ephemeral plants
*Eudicots*	*Brassicales*	*Brassicaceae*	*Erophila verna*	Ephemeral plants
*Eudicots*	*Brassicales*	*Brassicaceae*	*Hymenolobus procumbens*	Ephemeral plants
*Eudicots*	*Brassicales*	*Brassicaceae*	*Sisymbrium loeselii*	Ephemeral plants
*Eudicots*	*Caryophyllales*	*Caryophyllaceae*	*Cerastium semidecandrum*	Ephemeral plants
*Eudicots*	*Caryophyllales*	*Caryophyllaceae*	*Holosteum glutinosum*	Ephemeral plants
*Eudicots*	*Caryophyllales*	*Chenopodiaceae*	*Atriplex tatarica*	Annual herbs
*Eudicots*	*Caryophyllales*	*Chenopodiaceae*	*Bassia hyssopifolia*	Annual herbs
*Eudicots*	*Caryophyllales*	*Chenopodiaceae*	*Climacoptera crassa*	Annual herbs
*Eudicots*	*Caryophyllales*	*Chenopodiaceae*	*Halocnemum strobilaceum*	Long-lived perennial herbs
*Eudicots*	*Caryophyllales*	*Chenopodiaceae*	*Petrosimonia brachiata*	Annual herbs
*Eudicots*	*Caryophyllales*	*Chenopodiaceae*	*Petrosimonia oppositifolia*	Annual herbs
*Eudicots*	*Caryophyllales*	*Chenopodiaceae*	*Suaeda acuminata*	Annual herbs
*Eudicots*	*Caryophyllales*	*Frankeniaceae*	*Frankenia hirsuta*	Subshrubs
*Eudicots*	*Caryophyllales*	*Limoniaceae*	*Limonium caspium*	Long-lived perennial herbs
*Eudicots*	*Caryophyllales*	*Limoniaceae*	*Limonium scoparium (*L. *meyeri)*	Long-lived perennial herbs
*Eudicots*	*Caryophyllales*	*Plumboginaceae*	*Psylliostachys spicata*	Annual herbs
*Eudicots*	*Caryophyllales*	*Tamaricaceae*	*Tamarix laxa*	Shrubs
*Eudicots*	*Caryophyllales*	*Tamaricaceae*	*Tamarix octandra*	Shrubs
*Eudicots*	*Gentianales*	*Rubiaceae*	*Galium aparine*	Ephemeral plants
*Eudicots*	*Sapindales*	*Nitrariaceae*	*Tetradiclis tenella*	Ephemeral plants
*Monocots*	*Poales*	*Poaceae*	*Hordeum leporinum*	Ephemeral plants
*Monocots*	*Poales*	*Poaceae*	*Aeluropus littoralis*	Long-lived perennial herbs
*Monocots*	*Poales*	*Poaceae*	*Anisantha tectorum*	Ephemeral plants
*Monocots*	*Poales*	*Poaceae*	*Apera interrupra*	Ephemeral plants
*Monocots*	*Poales*	*Poaceae*	*Bromus japonicus*	Ephemeral plants
*Monocots*	*Poales*	*Poaceae*	*Bromus squarrosus*	Ephemeral plants
*Monocots*	*Poales*	*Poaceae*	*Eremopyron orientale*	Ephemeral plants
*Monocots*	*Poales*	*Poaceae*	*Eremopyron triticeum*	Ephemeral plants
*Monocots*	*Poales*	*Poaceae*	*Phleum paniculatum*	Ephemeral plants
*Monocots*	*Poales*	*Poaceae*	*Puccinellia gigantea*	Long-lived perennial herbs

### 1.2 Experimental design, materials and methods

Before auger holes drilling, we described vegetation throughout the whole key sites along 30-m long transects located at a distance of 2 m from each other. The interval of geobotanical descriptions along transects was 2 m. In total, 345 descriptions were made which include the number and size of the dominant plants at the shrub tier, their projective cover, the plant species and total projective cover of the herb tier, the portion of bare land (Table S1). The Latin names of plant species were given according to [7].

Phylogenetic tree of species found at the key site was built on a basis of a phylogenetic tree using scripts and instructions from [2]. 7 species absent in an initial tree [7] (*Limonium caspium, Limonium scoparium, Petrosimonia oppositifolia, Phleum paniculatum, Puccinellia gigantea, Suaeda acuminata, Tamarix octandra* named according to [8]) were added to basal nodes of their families using scripts provided in [7].

Auger holes are drilled according to semi-regular grid with an interval of 1 to 5 m from each other. From auger holes, soil samples (200 – 300 g) were taken from a depth 0–5, 5–10, 10–20, 20–30, 30–50, 50–70, 70–100 cm to measure pH value in paste and electrical conductivity (EC) in soil:water extract 1:2.5 (Table S2) by standard techniques (Table 2).

**Table 2:** Methods of chemical analyses of soils.

Analyzed parameters		Method, condition
Soil	Bulk density	Gravimetry
	Moisture	Gravimetry
	Particle-size distribution	A laser diffraction technique and an Analysette 22 Nano Tech equipment (Fritsch, Germany) in samples pre-treated with 4% Na_4_P_2_O_7_[9]
	Elemental composition	X-Ray fluorescence spectrometery, a Spectroscan Max-GV X-Ray fluorescence spectrometer^1^ (Russia) and a Russian soil standard sample according to the methodological recommendation (M-049-П/16). In 3 samples, the measurements were performed in two replicates.

Total organic carbon	Radiocarbon dates	Accelerator mass-spectrometry (AMS). Graphitization was performed using an AGE-3 graphitization system (Ionplus). Graphite ^14^C/^13^C ratios were measured using the CAIS 0.5 MeV accelerator mass spectrometer. The sample ratios were compared to the ratio measured from the Oxalic Acid II (NBS SRM 4990C). The quoted uncalibrated dates have been given in radiocarbon years before 1950 (years BP), using the ^14^C half-life of 5568 years. The error is quoted as one standard deviation and reflects both statistical and experimental errors. The samples are marked by the IGAN_AMS_ index. All the radiocarbon dates obtained were calibrated according to Intcal13 calibration curve with the use of the Calib 7.1. program (http://calib.qub.ac.uk/calib/) [1,10].

Soil paste	pH	Potentiometry, Hanna Combo HI 98,130 (Germany)
1:2.5 soil:water extract	EC	Conductometry, Hanna Combo HI 98,130 (Germany)

1:5 soil:water extract	pH	Potentiometry, Hanna Combo HI 98,130 (Germany) [11]
	EC	Conductometry, Hanna Combo HI 98,130 (Germany)
	Basicity from carbonates and soda	Titration with 0.02М H_2_SO_4_[12]
	Ca^2+^ and Mg^2+^	Titration with EDTA [9]
	Cl^–^	Titration with AgNO_3_[8]
	К^+^, Na^+^	Flame photometry [9]
	SO_4_^2–^	Sulphates were calculated as a difference between the sum of cations (Ca^2+^ Mg^2+^, К^+^, and Na^+^) and the sum of measured anions (Cl^–^ and HCO_3_^–^)

1 Energy resolution 9 eV (Si Kα), 60 eV (Fe Kα), X-ray tube power 160 W, Pd R-ray tube anode, crystal diffraction (LiF(200), C(002), PET, KAP (RbAP)).

At the typical microhigh and microlow, we dug two soil pits. Both pedons described are Gleyic Solonchaks (Loamic) according to [4]. From pits, soil samples (300 – 500 g) were collected from Ayz, Bgz and Cgz horizons from a depth 0 – 3 m. Radiocarbon dates for total organic carbon from the pedon located at microhigh (Table 3) were obtained as described in details in [10].

**Table 3:** The results of radiocarbon dating of soil samples (total organic carbon) from the pit located at the microhigh.

No IGAN_AMS_	Sampling depth, cm	^14^C age, BP (1σ)	cal BP
6200	0–10	235±20	68.3 (1 sigma) cal BP 157 - 165 0.255 285 - 301 0.745 95.4 (2 sigma) cal BP 0 - 5 0.029 152 - 169 0.330 281 - 307 0.641 Median Probability: 289

6201	12–18	3650±25	68.3 (1 sigma) cal BP 3920 - 3987 0.845 4048 - 4064 0.155 95.4 (2 sigma) cal BP 3892 - 4005 0.773 4032 - 4081 0.227 Median Probability: 3966

6202	25–35	3035±20	68.3 (1 sigma) cal BP 3183 - 3193 0.113 3208 - 3251 0.658 3303 - 3323 0.228 95.4 (2 sigma) cal BP 3170 - 3260 0.709 3288 - 3336 0.291 Median Probability: 3235

In soil samples taken from both pits (Table S3), we measured the moisture and soil bulk density, particle-size distribution, and elemental composition (total concentration of 18 chemical elements). We prepared a soil water extract (1:5 soil:water ratio) from samples collected from both pits to measure pH value (and in paste), basicity and the concentration of anions and cations.

The particle-size classes were defined according to recommendations of [4].

Statistical analyses included calculations of descriptive statistics for topsoils, subsoils and parent materials (Table S4).

Table S1. Description of vegetation in plots 2 × 2 m (4 m^2^) at the key site of 45 × 30 m

Table S2. Raw data for electrical conductivity and pH in soil samples collected from auger holes

Table S3. Raw data for soil proxies measured in samples collected from two soil pits

Table S4. Descriptive statistics

## Declaration of Competing Interest

All the authors declare that they have no known competing financial interests or personal relationships that could have appeared to influence the work reported in this paper.
